# Has the rheumatoid arthritis (RA) core outcome set influenced the selection of study outcome measures?

**DOI:** 10.1186/1745-6215-14-S1-O66

**Published:** 2013-11-29

**Authors:** Jamie Kirkham, Maarten Boers, Peter Tugwell, Mike Clarke, Paula Williamson

**Affiliations:** 1 University of Liverpool, Liverpool, UK; 2 VU Medical Center, Amsterdam, The Netherlands; 3 University of Ottawa, Ottawa, Canada; 4 Queen’s University Belfast, Belfast, UK

## Background

The development and application of standardised sets of outcomes to be measured and reported in clinical trials have the potential to increase the efficiency and value of research. One of the most notable of the current outcome sets was developed nearly 20 years ago: the World Health Organisation (WHO) and International League of Associations for Rheumatology (ILAR) core set of outcomes for rheumatoid arthritis clinical trials, originating from the OMERACT (Outcome Measures in Rheumatology) Initiative.

## Methods

A review of 350 randomised trials for the treatment of rheumatoid arthritis identified through *The Cochrane Library.* Reports of these trials were evaluated to determine whether or not there was a trend in the proportion of studies reporting on the full set of core outcomes over time. Researchers who conducted trials after the publication of the core set were contacted to assess their awareness of it and to collect reasons for non-inclusion of the full core set of outcomes in the study.

## Results

This review suggests that 60-70% of trialists conducting trials in rheumatoid arthritis are now measuring the rheumatoid arthritis core outcome set. 90% of trialists that responded said that they would consider using the core outcome set in the design of a new study.

## Conclusions

This review suggests that a higher percentage of trialists conducting trials in rheumatoid arthritis are now measuring the rheumatoid arthritis core outcome set. Core outcome sets have the potential to improve the evidence base for health care.

